# Re: Ethical issues in neurosurgery – A scoping review from the EANS ethico-legal committee. Brain Spine 2026: 105946

**DOI:** 10.1016/j.bas.2026.105985

**Published:** 2026-02-23

**Authors:** Tayfun Hakan

**Affiliations:** a University of Health Sciences, Hamidiye Faculty of Medicine, Department of Surgical Medical Sciences - Department of Neurosurgery, İstanbul, Turkey; b İstanbul Fatih Sultan Mehmet Training and Research Hospital, İstanbul, Turkey

**Keywords:** Education, Ethical governance, Morality, Neurosurgery, Professional ethics

Dear Editor,

I read with great interest the recent article published in Brain and Spine by Westarp et al. (2026), in which the authors provide a comprehensive review of ethical issues in neurosurgery, highlighting how ethical dilemmas may be classified differently across practitioners and subspecialties. Their contribution merits recognition for drawing attention to this important issue. The observation, noted in the limitations section, that what one author defines as an “ethical issue” may not be similarly classified by another is particularly thought-provoking. That such variation exists even among neurosurgeons who share comparable training and a common scientific language underscores the contextual and interpretive dimensions of professional ethics.

This plurality, however, invites careful conceptual reflection. A distinction may be drawn between morality and professional ethics. Morality concerns fundamental judgments about right and wrong—prohibitions against deliberate harm, deception, exploitation, or injustice. Professional ethics, by contrast, consists of articulated norms and codified standards intended to regulate conduct within a defined field. Ethics committees exist as institutional bodies; morality, by contrast, cannot be delegated in the same manner. Professional ethics can be formally structured, reviewed, and revised; moral responsibility, however, cannot be institutionalized in the same way, as it ultimately resides in persons. Professional codes of ethics are generally designed to regulate membership standards, professional competition, and safeguard the service ideals upon which professional legitimacy depends. One of the critical aspects of professional ethics is that individuals working in the same profession, regardless of their location, are expected to adhere to these codes of conduct.

The existence of ethical codes should not be equated with the guarantee of ethical practice. Unlike technical clinical guidelines, ethical norms do not operate mechanically. They require interpretation, internalization, and moral discernment. To assume that the mere formulation of rules will ensure their consistent application may reflect an overly procedural understanding of professional behavior. Human motivation, institutional pressures, and contextual complexities inevitably influence how norms are lived in practice.

The differentiation of ethical frameworks across general neurosurgery, pediatric neurosurgery, functional neurosurgery, and other domains reflects legitimate contextual needs. Yet the multiplication of categories should not obscure a central point: professional variation does not dissolve foundational moral commitments. Ethical norms may be specialized; the obligation not to harm, mislead, or unjustly disadvantage a patient remains constant.

Professional ethics, if confined to procedural rules, tends to regulate conduct without necessarily engaging the substance of ethical judgment—namely, value-based discernment. In Albert Camus's allegorical novel *The Plague (La Peste)*, Dr. Rieux faithfully performs his clinical duties by caring for his patients; at the same time, he undertakes an additional responsibility by pressing the authorities to acknowledge that the outbreak is the plague. This effort exceeds the routine expectations of professional role morality and reflects a commitment that is both ethical and civic. The example suggests that professional codes are not merely frameworks for correct performance of duty, but are closely intertwined with moral values that guide responsible action in times of crisis. For this reason, ethical governance in neurosurgery may require more than classification and codification. Just as clinical guidelines are developed through structured consensus processes, transparent methodologies, and periodic revision, the formulation and implementation of ethical standards may benefit from comparable rigor. Ethical norms are indispensable; precisely because of their importance, both their development and application demand sustained collective reflection and institutional attentiveness.

Professional expertise does not automatically confer moral infallibility. Ethical systems ultimately depend on the character and judgment of those who apply them. Strengthening ethical practice, therefore, involves not only drafting standards but also cultivating a professional culture in which foundational moral commitments are actively reinforced.

The authors’ analysis ultimately invites a deeper question: how can ethical standard-setting in neurosurgery move beyond codification and foster a sustained, methodologically rigorous engagement with moral responsibility?

## Funding sources

This research did not receive any specific grant from funding agencies in the public, commercial, or not-for-profit sectors.

## Declaration of competing interest

The author declares that he has no known competing financial interests or personal relationships that could have appeared to influence the work reported in this paper.
